# Correction to ‘Temporally balanced selection during development of larval Pacific oysters (*Crassostrea gigas*) inherently preserves genetic diversity within offspring’

**DOI:** 10.1098/rspb.2022.0153

**Published:** 2022-02-09

**Authors:** E. Durland, P. De Wit, C. Langdon

*Proc. R. Soc. B* **288**, 20203223. Published online 1 September 2021. (doi:10.1098/rspb.2020.3223)

In the Methods (section (d)), we state that initial samples of fertilized eggs were taken approximately 1 h after fertilization. This is inaccurate: samples were obtained approximately 5 h after fertilization, once embryos from *n* = 95 individual crosses had been pooled.

This correction does not affect any of the analyses, results or conclusions presented in the paper.

